# Bridging brain and body: a 3R framework for real-time self-regulation strategies in sport

**DOI:** 10.3389/fpsyg.2026.1833469

**Published:** 2026-06-12

**Authors:** Anastasia Yakushina, Nemanja Lakicevic, Sergey Leonov, Sergey Vvedenskiy, Patrik Drid

**Affiliations:** 1 Faculty of Psychology, Lomonosov Moscow State University, Moscow, Russia; 2 Federal Scientific Center of Psychological and Interdisciplinary Research, Moscow, Russia; 3 Institute of Natural Sciences and Sports Technologies, Moscow City University, Moscow, Russia; 4 Faculty of Sport and Physical Education, University of Novi Sad, Novi Sad, Serbia

**Keywords:** 3R framework, breathwork, meditation, self-regulation strategies, self-talk, sport psychology, visualization

## 1 Introduction

A notion that what separates elite athletes from the average ones is the mindset is widely accepted in the athletic community. The word “mindset” is very individual and has a different meaning for different athletes, but it can be derived that the mindset is determined and driven by the goals previously set. Though, the question that arises is if winners and losers have identical goals, that is, to win a particular competition or a championship, what are the decisive factors that determine who is successful and who is not? Indeed, training load and effort *per se* seem not to be a problem in achieving success once athletes are fully committed to a given sport, especially at the elite level. What seems to push elite athletes to continually advance in their careers is finding motivation to move forward and looking “beyond the horizon” whereby new goals can be identified and strived for. Yet, setting goals can also be problematic. Despite being useful at determining direction, reaching goals can lead to only a temporary improvement, can restrict content with the one's current career and can be at odds with the long-term progress (Clear and Clear, 2018). The alternative viewpoint on continual improvement in athletics is to focus on systems, i.e., methods to incrementally improve daily. Thus, daily practices that can be immediately implemented and require no sophisticated equipment can be of immense importance and utility to athletes both during practice and competition to reach peak performance when needed (Kharitonova and Klimova, 2025).

Not deviating from the path of daily improvement and “staying in the right lane” requires mental fortitude that turns many daily habits into automaticity, i.e., discipline which ultimately turns into expertise within a given sport. By definition, expertise “refers to the characteristics, skills, and knowledge that distinguish experts from novices and less experienced people” (Ericsson et al., 2006). Elite athletes (those competing at the highest level of their sport, i.e., Olympic and international level) may perceive and think in ways that differ meaningfully from those of average athletes. Exploring metacognition, that is, thinking about thinking, can help scientists and practitioners greatly in understanding how thinking is formed and executed at this level of athletic competition. The term metacognition has been initially described nearly 50 years ago as one's insight into and control over their own mental processes (Flavell, 1979). Expertise in sport is closely related with metacognition in both training (e.g., knowledge of when a skill has been acquired) and competitive settings (e.g., self-regulation under stress) (Ericsson et al., 2006). Metacognition can help athletes observe their current state (self-monitor performance and internal states) and adjust accordingly to attain goals previously set and as such is closely linked to self-regulation (Morosanova et al., 2026). From a psychological perspective, self-regulation is defined as the “self's capacity to alter its responses, including thoughts, emotions, and behaviors, in accordance with standards or goals” (Baumeister et al., 2007; Prokhorov et al., 2026). Given that elite-level athletes aim to continually improve, they might relate better to the developmental definition of self-regulation which defines it as “the ability to manage one's behavior, emotions, and thoughts in pursuit of long-term goals” (Blair and Ursache, 2011). Well-developed self-regulation is of great importance for athletes as they regularly need to deal with anxiety ahead of the competition, focus during high-stakes moments and refrain from emotional reactions that might impede performance (Jonker et al., 2010; Molodozhnikov and Guseinov, 2024). In training, self-regulation helps athletes adhere to training plans, maintain effort during fatigue and persist through setback and plateaus (Toering et al., 2009; Klimanova et al., 2026). As elite athletes seek continual improvement, they use self-regulation during learning and skill-acquisition, during reflection on performance feedback and while adjusting techniques and engagement in deliberate practice (Zimmerman and Kitsantas, 2005).

In recent years, a growing number of elite-level athletes have spoken openly in interviews and post-game discussions about non-physical self-regulation techniques they incorporate into their daily routines to sharpen their mindset. These strategies—ranging from visualization and mindfulness to breathing exercises, meditation, self-talk, and structured pre-performance rituals—are increasingly highlighted as essential components of their overall preparation (Bühlmayer et al., 2017). Many athletes now attribute a substantial part of their success to these practices, often suggesting that the mental dimension of training holds equal, if not greater, importance than the physical work performed on the field or in the gym. These approaches seem logically sound and theoretically justified given that the main interaction between metacognition and self-regulation is to control, monitor, and regulate strategies to meet task demands and achieve goals (Tarricone, 2011). In the following paragraphs, we will discuss some of the most claimed self-regulation techniques used by elite athletes, their scientific merit and future perspectives. We will also introduce the new proposed conceptual framework 3R—recognize, regulate, and refocus, which places self-regulation as the principal determinant of performance that gives athletes ability to readily apply these techniques regardless of the context.

However, existing models of self-regulation in sport share two common limitations when applied to real-time competition. First, they are predominantly descriptive or phase-oriented (forethought—performance—self-reflection), which works well for practice and long-term development but does not specify how an athlete should intervene within seconds during a penalty shot or a break point. Second, most models treat regulatory strategies as interchangeable tools without clarifying the necessary pre-conditions (accurate recognition) and post-conditions (attentional refocusing) for their effective use. Consequently, athletes often learn what to do (e.g., self-talk) but not when, why, or how to transition out of the technique.

The primary aim of this opinion paper is therefore twofold. First, we provide a concise synthesis of commonly used self-regulation techniques anecdotally reported by elite athletes, grounding each in contemporary empirical evidence (if available). Second, and more importantly, we propose the 3R Framework—Recognize, Regulate, Refocus—as an integrative model that explains how these techniques can be deployed dynamically in real time. By embedding practical strategies within a unified metacognitive framework, we seek to enhance their applicability, transferability, and effectiveness across sporting contexts.

## 2 Approach to literature selection

As this is an opinion paper, we did not conduct a formal systematic review. Instead, we adopted a purposive narrative synthesis guided by three criteria: (a) peer-reviewed empirical studies or meta-analyses examining the effects of visualization, self-talk, breathwork, or meditation on sport-related outcomes; (b) sources published in English within the last 15 years, with priority given to recent meta-analyses (Liu et al., 2025; Laborde et al., 2024); and (c) triangulation with publicly reported practices of elite athletes to ensure ecological validity. Selection bias was minimized by cross-referencing cited literature across multiple key sources (Hatzigeorgiadis et al., 2011; Bühlmayer et al., 2017). The resulting synthesis is not exhaustive but illustrative, intended to ground the 3R framework in existing evidence while acknowledging gaps.

## 3 The 3R framework of self-regulation

Professional athletes face immense pressure to succeed from both outside (coaches, peers, parents) and within (self-imposed). Maintaining a path of continual athletic improvement requires meticulous psychological care that athletes are often missing (Lane, 2024). In the absence of professional help (or even when it exists), practical, scientifically grounded, zero-cost, and easy-to-do tools can be of great importance and utility to athletes. For this reason, we sought to introduce the 3R Framework which conceptualizes self-regulation as a cyclical, real-time process consisting of three interrelated stages: recognize, regulate, and refocus. The framework is grounded in metacognitive theory, stems from amounting scientific evidence and aligns with principles of physiological and psychological homeostasis.

### 3.1 Recognize

Recognition refers to the athlete's ability to accurately perceive and label their current internal state (Liao and Masters, 2002). This includes cognitive (e.g., attentional focus, thought patterns), emotional (e.g., anxiety, frustration, confidence), and physiological (e.g., arousal, muscle tension, breathing patterns) dimensions. Put simply, recognition enables athletes to comprehend what is happening within the boundaries of their skin. It constitutes a prerequisite for any deliberate regulatory intervention, as the athlete directs attention inward to become aware of their ongoing processes. Without accurate recognition, regulatory attempts risk being mismatched or ineffective.

The effectiveness of recognition allows for the precise application of strategies relevant to the identified state. Furthermore, high sensitivity to early signals of destabilization (e.g., minimal cognitive distraction or muscular tension) establishes a foundation for proactive, preventive self-regulation, which is a hallmark of expert-level proficiency (Gröpel and Mesagno, 2019). Moreover, a well-developed capacity for recognition not only optimizes the selection and implementation of regulatory techniques but also enhances the athlete's perceived control and self-efficacy, which ultimately builds athletes' self-confidence.

### 3.2 Regulate

Regulation involves the intentional modification of the recognized state to better align with task demands. This process is implemented through the selective application of specific techniques aimed at correcting identified imbalances in the affective, cognitive, or somatic domains. This may include downregulating excessive arousal, increasing activation, restructuring maladaptive thoughts, or stabilizing attention (Jonker et al., 2010; Ong and Griva, 2017). The selection of specific regulatory strategies is determined by a complex of contextual factors, including the specifics of the sporting task, time constraints, and the individual psychological characteristics of the athlete (Latinjak, 2025).

### 3.3 Refocus

Refocusing, the final stage, serves as a necessary cognitive transition that completes the self-regulation cycle. It refers to the redirection of attention toward task-relevant cues following regulation. Its key function is to shift focus from internal regulatory processes to external, task-relevant elements and specific, actionable goals (e.g., the technique of the next element, a tactical cue). This stage ensures that regulatory efforts translate into improved performance rather than inwardly focused rumination (Latinjak, 2025). Thus, refocusing acts as the mechanism for operationalizing the results of regulation. It grounds the athlete in the present moment and facilitates the translation of an adjusted psychophysiological state into optimal, goal-directed behavior, which constitutes the ultimate objective of the entire self-regulation system.

## 4 3R techniques

### 4.1 Visualization

Likely the most commonly discussed non-physical technique elite athletes report using on a daily basis to excel their performance in visualization. For athletes, visualization or self-imagery simply refers to the mental rehearsal or visualization of skills and scenarios to improve performance, confidence, and focus when it matters the most (Sheikh and Korn, 1994). Through years, legends in their respective sports such as Novak Djokovic, Cristiano Ronaldo, Kobe Bryant, Connor McGregor and others have extensively talked about all the perceived benefits they obtain from visualization. In 2019, Djokovic said that during visualization he always imagines himself as a winner and that he plays the match in his head before he steps on the court. Ronaldo recently stated visualization helps him immensely prepare for the upcoming game while Connor McGregor (mixed-martial arts fighter) commonly contributes his victories to visualization: “you can visually imagine your entire world. If you can see it here (point to his head) and you have a courage to speak it. It will happen. I see these shots. I see these sequences and I don't shy away from them.” Michael Phelps who won 8 gold medals at the Olympic Games in China in 2008 claimed that visualization helps him prepare for the best and the worst scenarios that might occur. Prior to joining the NBA, Kobe Bryant spoke extensively about his visualizations: “I would imagine playing for the Lakers, I would imagine what the uniforms look like, I would imagine us playing and the smell of the arena.”

Recent evidence on visualization in sports shows that athletes with higher athletic achievement tend to have stronger imagery abilities (Volgemute et al., 2025). In particular, visualization has been found to be especially helpful in tennis and football by enhancing athletic performance, including agility, and muscle strength (Liu et al., 2025). Available evidence suggests that the effectiveness of visualization training depends on its frequency, duration and consistency. Regular but adjustable imagery sessions kept within 30 to 40 min, at least two sessions per week, can effectively enhance athletic performance by reinforcing neuromuscular pathways and cognitive processes without causing cognitive fatigue (Murphy, 2012). In addition, a large meta-analysis on the effectiveness of visualization in sports found that practice can also be divided into shorter sessions of 10 min, 3 times per week over a span of 100 days, which produced the strongest performance gains in athletes (Liu et al., 2025). Athletes reported also using visualization techniques learned after intervention in studies outside of the athletic context to plan and manage thinking (Rhodes et al., 2024). Neuroscientists argue that visual and kinesthetic visualization share similar neural networks leading to the conclusion that merged interventions are beneficial to athletes whereas separate use of those two modalities of imagery may seem less efficient (Filgueiras et al., 2018). Now, what individual athlete chooses to visualize is dependent on their personal preference, given context and guidance that they receive from their coaches and/or sport psychologist. Some of the techniques discussed among athletes is breaking down a complex skill or routine into smaller steps and mentally practice each one with perfect execution and accuracy, focusing on successful outcomes such as scoring a goal, crossing the finish line first. hitting a home run, making a free throw, etc., and imagining different scenarios (how to respond to various game situations or challenges, including handling mistakes, managing pressure, or dealing with unexpected events).

### 4.2 Self-talk

Self-talk is commonly used among elite athletes. Self-talk can be defined as an inner dialogue whereby the sender of a self-talk message is also the intended receiver (Van Raalte et al., 2016). Over the years, it has been endorsed by coaches and athletes as one of the most widely used and effective strategies for enhancing sport performance (Thelwell et al., 2008; Vargas-Tonsing et al., 2004). Self-talk may enhance athletic performance by improving attention control (“stay focused”), cognitive restructuring (“focus on the opponent”), motivation regulation (“keep pushing”), and emotional control (“stay calm, breathe slowly”) (Hatzigeorgiadis et al., 2009; Hatzigeorgiadis and Galanis, 2017).

Put simply, it can be divided into negative and positive self-talk and what seems to set elite athletes apart once again is going against the human nature and our proneness to engage in negative self-talk and emphasizing positive side of it. It works by adjusting one's current state to the one necessary to achieve predefined goals. Athletes deem it particularly useful to break the pattern of negative thinking and reframe their minds toward goal-oriented behavior.

Over the recent years, public has witnessed athletes utilizing self-talk on some of the highest sport stages in the world. Like in the case of visualization, Ronaldo is a loud advocate of using self-talk to achieve success: “I speak to the ball. I tell it, ‘Do the right trajectory; go to the net.' Kylian Mbappé, a fellow football player and one of the best active football players in the world also uses self-talk: “Every time I step on the field, I say to myself I am the best in the world, despite being out there with Messi and Cristiano. You do not give yourself any limits.” In a similar manner, George Kittle, an elite-level American football player has been caught on camera numerous times engaging in self-talk: “you are great, you are healthy you are strong… breathe in confidence, exhale fear, breathe in belief, exhale doubt.” Self-talk is also commonly found among elite athletes in individual sports. For instance, Floyd Mayweather, arguably one of the best boxers of all time frequently engages in self-talk:” Nobody can beat me. There is no fighter better than me. Put them in front of me, I am going to beat them.” Serena Williams who won 23 Grand Slam singles titles in tennis, credits her success to mental game, including self-talk as she frequently used sentences such as: “I am strong. I am a champion.” Similarly, Jasmine Swawyers, a 2023 European indoor long jump champion was recorded ahead of the jump whispering to herself: “This is it. Drive down into the track. Keep running. Sprint.”

A meta-analysis of 32 individual studies yielding 62 effect sizes found that interventions including self-talk training were more effective than those not including self-talk training (Hatzigeorgiadis et al., 2011). This technique was more effective for tasks involving relatively fine, compared with relatively gross, motor demands, and for novel, compared with well-learned, tasks. Instructional (how to execute a particular task) self-talk was more effective for fine tasks than was motivational self-talk; moreover, instructional self-talk was more effective for fine tasks rather than gross tasks. Though, research on self-talk in sports is at its infancy, practical examples of elite athletes using it are abundant.

### 4.3 Breathwork

At the fundamental level, breathwork can be defined as the conscious control of breathing to influence mental, emotional, and physical state (Fincham et al., 2023). The practice of conscious breathing is not anything new. This practice is deeply rooted in the traditions of Eastern cultures (especially India) where the potency of it was recognized long time ago. In recent times, breathwork practice was popularized by people like Wim Hof, also known as the Iceman, who combined deliberate cold exposure with breathing techniques to embark on the quests such as climbing Kilimanjaro in shorts and running a marathon in the Death Valley, California without a drop of water. Nowadays, promising effects of the Wim Hof method of conscious breathing and deliberate cold water exposure to enhance health, performance and recovery are being studies in laboratories all around the globe (Almahayni and Hammond, 2024; Ketelhut et al., 2023; Marko et al., 2022; Mullooly and Colbert, 2024).

World's top athletes are quite transparent about deliberate breathing. One of the best shooters in the NBA history, Stephen Curry recently stated: “Breathing is a skill. I have tried to master breathing over the last five of 6 years.” Once again, Djokovic emphasized his breathing technique: “Learning how to breathe consciously is one of the biggest things you can do for your mental health, your recovery, and your presence in the moment.” An elite level football player, Jorge Luiz Frello Filho, claim his calm during penalty kick to deep breathing: “I always do deep breaths before the penalty kick. It gives me confidence.” Also, Justin Fields, one of the young prospects in American football recently stated: “I do this breathing exercise, 4 s in 6 s out and it really helps me to stay calm. I also don't get as tired as well.”

In the beginning, breathwork was disregarded by the scientific community as not being scientifically sound and without evidence, but more and more studies are being published showing that breathwork can be of great utility in exceling health and performance in both athletes and non-athletes alike (Almahayni and Hammond, 2024; Blades et al., 2024; Ketelhut et al., 2023). There are a number of variations of breathwork many of which stem from yoga. Some of the most known are diaphragmatic (belly breathing), box breathing, 4–7–8 breathing, alternate nostril breathing, pranayama, yoga nidra (also known as non-sleep deep rest) and others (Zaccaro et al., 2018). Depending on the context and the current state, these techniques are most commonly used to relax and calm the mind down by emphasizing parasympathetic activity of the autonomic nervous system. To date, the most researched breathwork techniques in athletes are slow paced breathing and breath-holding (Laborde et al., 2024). Findings indicate that slow paced breathing and breath holding were related to improved performance in sports, with large and small effect sizes for longer-term interventions, respectively, while in short-term interventions, slow paced breathing, breath holding, and voluntary hyperventilation were unrelated to sports performance. However, 4-week supplemental breathing protocol did not produce aerobic performance of recreational runners (Wolff et al., 2025). Importantly, breathwork techniques such as deep diaphragmatic breathing provide enhanced feeling of relaxation in athletes, and oftentimes this can be crucial in the moments of tension, especially during major competitive events (Hunt et al., 2018). Thus, breathwork is a skill-based tool that can be used at any venue, in any context and can be performed in combination with visualization and/or self-talk to reap the most benefits. Its exact application depends predominantly on the situation an athlete is in at a given moment, his/her preference and what effect are their seeking for (fast-paced breasting—excitatory, slow-paced breathing—inhibitory, i.e., calming).

### 4.4 Meditation

Just like in the case of breathwork, meditation in an ancient practice, also originating in the Far East cultures. There is no universally accepted definition of meditation, as currently there is more than 300 variations of it (Matko and Sedlmeier, 2019). In the 1970s, Davidson and Coleman defined meditation as rather a field of practice that refers to a wide variety of techniques and approaches (Davidson and Goleman, 1977). In the literature, descriptions such as “sustained, focused awareness in which the mind rests steadily on a single object, principle, or state of being.” and “meditation is the continuous, disciplined attention of the mind.” are commonplace. Jon Kabat-Zinn, who was a molecular biologist at the Massachusetts Institute of Technology, is arguably the single most important figure for mainstreaming meditation in secular contexts. He pioneered a concept of Mindfulness-Based Stress Reduction (MBSR) in 1979 thereby stripping Buddhist meditation of its religious elements and framing it as a scientific, evidence-based practice for pain management and stress. He opened the door for its adoption in thousands of hospitals, clinics, and research institutions worldwide. Later, in the early 21st century, researcher and author, Dr. Joe Dispenza, was and still is one of the most prominent advocates for implementing meditation into daily lives of everyday people with his research group publishing some groundbreaking studies in the last several years (Jinich-Diamant et al., 2025; Stapleton et al., 2024; Zuniga-Hertz et al., 2025). As meditation becomes more mainstream in sports, one of the best players in the history of NBA, Lebron James, is on a commercial for a popular meditation app—Calm. It is not uncommon to see James during games, on the sideline or on the bench with his eyes closed presumably practicing a breathwork. This does not come as a surprise as he is a great admirer of Kobe Bryant who claims that he meditates every morning for about 15 min as he believes that this sets gim up for the rest of the day. He further stated that meditation is “anchor” for him and when he does not do it he feels as he is constantly chasing the day as opposed to dictating the day.” In a similar fashion Djokovic claimed that daily meditation is really important to him as it helps him perform on and off the court.

As power of meditation is reported in many other elite athletes as the message is being spread constantly and scientific evidence on the benefits of meditation in athletes accumulates. A recent meta-analytical review showed that physiological and psychological surrogates improved to a meaningful extent following mindfulness (meditation) practice, as well as performance outcomes in shooting and dart throwing (Bühlmayer et al., 2017). In addition, meditation reduces symptoms of anxiety and stress, and improves psychological wellbeing in athletes (Myall et al., 2023). Regular meditation positively impacts elite athletes' psychological state, enhancing mindfulness, resilience, flow, and alleviating anxiety, depression, and psychological fatigue, but not subjective wellbeing (Zhang et al., 2025). The exact prescription on how frequent and how long meditation sessions in athletes ought to be the reap the benefits remains a matter of debate given the individual variations in meditative experience. Nevertheless, available evidence points to the goal of accumulating at least 60 min of meditation weekly in a single or multiple sessions (Zhang et al., 2025).

## 5 Final remarks

The 3R Framework offers a unifying model for understanding how diverse self-regulation techniques function within a coherent metacognitive process. Rather than prescribing specific tools, the framework emphasizes adaptability, situational awareness, and intentional choice. This approach aligns with the realities of elite sport, where contexts and performance demands fluctuate rapidly and rigid routines may fail. By foregrounding recognition and refocus, the framework addresses common limitations in applied practice—namely, misapplication of techniques and excessive inward attention. Importantly, the model is scalable across expertise levels and adaptable to individual differences.

Based on the context they are in, athletes can choose any of the above-mentioned techniques to enhance their performance. The idea is for athletes to have a “portable toolbox” that in every situation gives them ability to recognize internal states (e.g., anxiety, distraction, frustration), regulate these states using psychological skills listed above and refocus attention on task-relevant cues. However, athletes should also be aware of potential pitfalls of self-regulation that can in certain situations impair their performance. Excessive self-monitoring can backfire as over-awareness may disrupt automatic motor execution, a phenomenon also known as “paralysis by analysis” under pressure. Also, if mediation is misapplied, it may reduce competitive intensity in athletes who require high arousal. In a similar manner, overuse of breathwork might trigger under-arousal in explosive sports. Likewise, ineffective visualization may reinforce fear-based scenarios. Collectively, this can lead to mental fatigue and cognitive overload. Thus, if they decide, athletes should receive 3R prescription from sport psychologist that is based on their unique demands to produce desired effects.

Unlike stage-based model (Zimmerman, 2000) that emphasize forethought and self-reflection across longer time scales, the 3R Framework is designed for within-task regulation. Compared to control theory (Carver and Scheier, 1982), which focuses on discrepancy reduction, 3R explicitly addresses the qualitative shift from internal monitoring (Recognize) to external action (Refocus). Relative to recent integrative proposals (Latinjak, 2025), our framework does not prescribe specific techniques but rather provides a metacognitive sequence that enhances technique selection and timing. Thus, 3R is complementary rather than competitive—it fills the real-time execution gap.

## 6 Limitations

The present opinion paper is subject to several important limitations that should be acknowledged. First, the proposed framework originates primarily from extensive practical experience working with athletes, many of whom have consistently highlighted the increasing psychological and performance-related demands of professional sport. While this applied perspective provides valuable ecological validity and real-world relevance, it also introduces a degree of subjectivity. The insights that informed the development of the 3R framework are shaped by practitioner observation and athlete self-report, rather than being derived exclusively from controlled empirical investigation.

Second, the process used to identify and incorporate relevant literature was not fully systematic. Although the inclusion of sources was guided by clear criteria—namely, relevance to the topic and publication in peer-reviewed journals—the search and selection procedures did not follow a formal systematic review protocol. As a result, there is a possibility of selection bias, and some pertinent studies may have been unintentionally omitted. This limits the comprehensiveness and reproducibility of the review process and means that the conclusions drawn should be interpreted with caution.

Furthermore, the paper adopts a predominantly narrative approach, synthesizing findings and practical insights in a way that prioritizes conceptual development over exhaustive evidence aggregation. While this allows for flexibility and the integration of multidisciplinary perspectives, it also means that the strength of evidence supporting specific components of the 3R framework may vary. Not all elements have been equally validated through rigorous experimental or longitudinal research, highlighting the need for future empirical studies to test and refine the framework.

Finally, the applicability of the proposed framework may be somewhat constrained by the population from which it was derived. The focus on top-tier and elite athletes means that the strategies and principles outlined may not directly generalize to other populations, such as recreational athletes or individuals in non-sport performance domains. Future research should aim to examine the transferability and effectiveness of the 3R framework across diverse contexts and levels of expertise.

Taken together, these limitations underscore that the present paper should be viewed as a theoretically informed and practice-driven contribution, rather than a definitive or exhaustive account. It is intended to stimulate further research, discussion, and empirical validation of the proposed ideas.

## 7 Conclusion

Elite athletic performance depends on the ability to self-regulate internal states in real time. Visualization, self-talk, breathwork, and meditation are valuable tools, but their effectiveness is maximized when embedded within a coherent regulatory framework. The 3R framework—recognize, regulate, refocus—offers a parsimonious, evidence-informed model that integrates metacognition, self-regulation, and applied practice. By shifting emphasis from isolated techniques to adaptive processes, the framework provides a foundation for both performance enhancement and long-term athlete development. For practitioners, the 3R Framework can inform assessment, intervention design, and athlete education. Coaches and sport psychologists may use the framework to: (1) diagnose self-regulation breakdowns; (2) teach athletes when to use techniques, not just how and (3) foster autonomy and metacognitive skill development.
